# Seasonal variation in meat quality of Angus steers raised in a Mediterranean forage-fed system: A farm case study

**DOI:** 10.1371/journal.pone.0344517

**Published:** 2026-03-24

**Authors:** Viviana Bolletta, Valentina Roscini, Emanuele Lilli, Chiara Fodaroni, Jacopo Gabriele Orlando, Valentino Mercati, Bernardo Valenti, Mariano Pauselli

**Affiliations:** 1 Dipartimento di Scienze Agrarie, Alimentari e Ambientali, University of Perugia, Perugia, Italy; 2 Aboca S.P.A. - Società Agricola, Arezzo, Italy; University of Naples Federico II: Universita degli Studi di Napoli Federico II, ITALY

## Abstract

This study monitored the impact of seasonal feeding variability on meat quality traits in forage-fed Angus steers. A total of 38 steers were selected based on their diets during the last three months before slaughter: a hay-based winter diet (WIN), a spring fresh-forage-based diet (SPR), or a summer fresh-forage-based diet (SMM). Meat quality was assessed through fatty acid profiling, antioxidant capacity, myoglobin content, color stability, and lipid oxidation during refrigerated storage. Principal components and linear discriminant analysis were used to uncover, underlying patterns, and classify the observations based on the multivariate profile. Meat fatty acids showed limited variation across seasons, with differences restricted to minor polyunsaturated fatty acids. The n-6/n-3 ratio and other health-related lipid indices remained within recommended values despite the feeding season. Meat from SPR and SMM animals showed higher *α*- and *γ*-tocopherol levels and greater myoglobin content compared to WIN meat. Color parameters and lipid oxidation were moderately affected by diet and storage time, with better stability observed in meat from animals receiving fresh forage. Despite the subtle impact on individual quality traits, multivariate discriminant analysis effectively differentiated meat samples based on the feeding season, achieving 89.5% correct classification. The most discriminating variables included fatty acids (17:1 c*9*, 18:1 *t*10, 22:0, 22:5 n-6), color attributes (b*), and oxidative markers (TBARS, metmyoglobin %). With the caution due to the nature of the study, these findings suggest that while single quality indicators may not strongly reflect dietary differences, their combination may provide a tool to trace seasonal feeding strategies in grass-fed beef production.

## Introduction

In recent years, consumer demand for grass-fed meat has constantly increased [1], as it is perceived as a healthier alternative to conventional meat [2]. In the present study, the term “grass-fed” refers to the traditional definition adopted before 2016 in the United States, indicating ruminants exclusively fed on grass and forage throughout life, with continuous access to pasture.

Animal diet is one of the major factors affecting the chemical composition and nutritional properties of meat [3–4]. Usually, as compared to meat from cattle fed a concentrate-based diet, grass-fed meat is leaner with a greater proportion of polyunsaturated fatty acids with anti-inflammatory, anti-cholesterolemic, and anti-carcinogenic properties, such as those of the n-3 series (18:3 c9c12c15, alpha-linolenic acid; 20:5 c5c8c11c14c17, eicosapentaenoic acid; 22:6 c4c7c10c12c16c19, docosahexaenoic acid) and rumenic acid (18:2 c9t11) [5–8]. Moreover, grazed pasture may serve as a natural source of phytochemicals with antioxidant potential, whose presence in meat is directly influenced by the animals’ diet. Van Vliet et al. [9] reviewed that terpenes, phenols, vitamins, and carotenoids increase in ruminant meat and milk along with the percentage of pasture in the diet and the botanical diversity of grazed pasture, thus improving the antioxidant capacity of the products.

However, the chemical composition of pasture undergoes variations throughout the year [10–13], due to both the phenological stage of the plants and the variable availability of plant species across the seasons [14]. These changes may affect the nutritive value of green forage in terms of macro- and micronutrients. Overall, the nutritive value of green forages decreases with plant senescence due to the reduction of protein percentage and the increase of non-degradable fiber. Similarly, older plants contain lower quantities of vitamins. Considering that the finishing diet may affect meat composition and quality, it is supposable that, in a grass-fed system, the above-described variability in the characteristics of the diet ingested by the animals in the months immediately before slaughter could determine variations in meat quality traits, which could represent a relevant issue to consider for grass-fed producers. So far, limited literature seems to support this hypothesis [15–16], and it refers mainly to non-Mediterranean regions, where both the grass-fed production system and its implications for meat quality have received little scientific attention.

In light of the above, this study aimed to assess meat quality traits from grass-fed steers reared under commercial farm conditions in a Mediterranean environment and slaughtered in different periods of the year corresponding to different diets during the last three months before slaughter, with a particular focus on meat fatty acids, color, antioxidant capacity, and resistance to oxidative degradation.

## Materials and methods

### Animals, diets, slaughter, and sampling

This study was conducted between 2020 and 2022 in the facilities of a commercial farm located in Tuscany region (Italy; 43.31484, 11.82710) and oriented towards the production of organic grass-fed meat. Approval by the Bioethics Committee of the University of Perugia was not required due to the survey-like nature of the study. All animals monitored during the observation period were not specifically reared and slaughtered for this study but rather for commercial meat production, following the standard practices of the farm. Nonetheless, the animals were handled and slaughtered by qualified personnel, following the current European regulations on animals designated for scientific research [17].

Fifty-two weaned Angus steers were purchased from the same supplier (where a grass-fed system was also adopted) and arrived at the farm between March and August 2020 at an average age of 362 days ± 42 and an average weight of 294 kg ± 52 (mean ± standard deviation). Since their arrival, the animals were fed according to the grass-fed system adopted by the farm. Specifically, during the grazing season (mid-March to mid-November), the animals grazed during the day and were moved to a paddock with a shelter for the night, where alfalfa, sainfoin, and ryegrass hay (one round bale per type) were always available. During the non-grazing season (mid-November to mid-March), the animals were kept in a sacrifice paddock with a night shelter for most of the time and fed the same hay as in the grazing season. In addition, during the non-grazing season, the animals were allowed to pasture whenever weather conditions permitted. No grains, concentrates or additives were ever provided to the animals.

The grazing area consisted of 30 fenced plots (average size 700 m x 40 m) seeded with multispecies meadow (*Avena sativa* 50%, *Lolium multiflorum* subsp. *Westervoldicum* 25%, *Trifolium alexandrinum* 10%, *Trifolium incarnatum* 5%, *Vicia sativa* 5%, *Vicia villosa* 5%), sainfoin, sudan grass, low-saponin alfalfa and *Lolium italicum*, or ryegrass. Grazing followed a strip-grazing model. The same plots were also used to produce the hay provided to the animals. Strip-grazing was implemented by subdividing each plot into subplots (40 x 20 m surface) using movable electric fences. The fences were moved when the residual herbage height reached 30 cm for sudan grass and 10 cm for the other species to ensure adequate regrowth. Pasture samples were collected from each sub-plot on the first day of grazing. Each round bale of hay offered to the animals was also sampled. All collected samples were frozen at −80°C, freeze-dried and stored at −30°C, pending analyses.

The diet consumed by each animal during the last months before slaughter was reconstructed retrospectively based on grazing duration on each plot, the stocking rate per plot, biomass availability, and hay intake. Based on these data, a subset of 38 animals consuming three different diets (winter, spring, and summer) during the last three months before slaughter was identified, ensuring comparable initial age and body weight across individuals. In detail, a hay-based diet consisting of hay (80%) and for the other part by grazed ryegrass (10%) and multispecies meadow (10%) was consumed by 13 animals and represented the winter diet (WIN); a pasture-based diet constituted of grazed sainfoin, alfalfa, lolium, and multispecies meadow (20% each) and for the other part by hay (20%) was consumed by 13 animals and represented the spring diet (SPR); another pasture-based diet consisting of grazed sudan grass, alfalfa, and lolium (65%, 7.5%, and 7.5%, respectively) and for the other part by hay (20%) was consumed by 12 and represented the summer diet (SMM). Table 1 reports the chemical composition of the three seasonal diets. Evaluation of meat quality parameters was performed only on the selected subset of 38 steers.

**Table 1:** Composition of the diet ingested by the animals during the last three months before slaughter.

	Feeding Season^1^		
Items	WIN	SPR	SMM
**Chemical composition, g/kg DM** ^**2**^			
**Crude Protein (CP)**	83.0	128.4	82.6
**Ether extract**	10.2	15.8	12.7
**NDF^3^**	653.4	479.1	543.0
**ADF^4^**	414.9	339.6	368.4
**ADL^5^**	52.8	50.3	47.3
**Hemicellulose**	238.7	140.9	175.1
**Cellulose**	362.8	288.1	321.7
**Ash**	84.8	80.1	66.7
**Protein Fractions**^**6**^ **(% CP)**			
**A**	16.6	16.8	16.9
**B1**	6.72	22.0	18.1
**B2**	34.2	39.6	33.7
**B3**	23.3	13.5	20.2
**C**	11.1	8.13	9.17
**Fatty acids (g/100g total fatty acids)**			
**14:0**	1.21	1.13	1.00
**16:0**	33.7	22.9	29.8
**18:0**	6.7	5.3	10.1
**18:1 c9**	5.8	3.4	5.1
**18:2 c9c12**	17.1	16.8	20.3
**18:3 c9c12c15**	31.0	47.6	31.4
**Phenols and tocopherols**			
**Total phenols^7^**	6.6	12.0	11.7
**Total tannins^7^**	1.8	1.9	5.2
**α-tocopherol (mg/kg DM)** **γ-tocopherol (mg/kg DM)**	0.4	1.7	2.1
	0.1	0.4	0.5

^1^Feeding season: WIN = Hay-based diet, with hay ≥ 80% total ingestion); SPR = Spring Pasture-based diet, with pasture ≥ 80% total ingestion diet; SMM = Summer Pasture-based diet, with pasture ≥ 80% total ingestion diet)

^2^DM: Dry Matter

^3^NDF: Neutral Detergent Fiber

^4^ADF: Acid Detergent Fiber

^5^Acid Detergent Lignin

^6^Protein Fractions: A = Non-Protein Nitrogen; B1 = buffer-soluble true protein; B2 = buffer – insoluble protein – neutral detergent soluble protein; B3 = neutral detergent insoluble protein – acid detergent insoluble protein; C = acid detergent insoluble protein

^7^Total phenols and tannins are expressed as mg of tannic acid equivalent per gram of DM

Animals were slaughtered once they reached the target commercial weight of 550–600 kg, ensuring that slaughter was based on weight rather than a fixed timeline. Within each group, slaughter was performed in 3 successive batches over a period of approximately one month, ensuring that all animals were processed shortly after achieving the target weight. For each individual, on the afternoon of the last day of permanence on the farm, the final live weight was recorded during loading onto the truck before moving the animal to a commercial abattoir 60 km away from the farm (transport duration about 1 h). On the following morning, the animal was slaughtered according to EU regulation. After recording hot weight, the carcass was halved and stored for 24 h at 4 °C. Then, the cold carcass weight was recorded, and the pH measured on the *Biceps femoris* muscle. The probe of a pH meter (Orion Star A111 pH meter, Thermo Scientific, Singapore) was inserted through a small incision made with a scalpel. The pH meter was calibrated with standard buffer solutions at pH 4.0 and 7.0. Finally, the carcass was aged during 21 days at 0–2 °C before meat sampling.

The *Biceps femoris* muscle was excised from the right half of the carcass and a portion (about 500 grams) between 25 cm and 50 cm distance from the insertion point was taken, vacuum packed and transferred under refrigerated conditions to facilities of the University of Perugia, where it was subdivided into aliquots, each assigned to a specific analysis ensuring that the same anatomical portion of the muscle was consistently used for the same analysis across all animals. Aliquots were stored at −80 °C pending analyses, except for color and lipid oxidation.

### Forage analyses

For each type of forage, samples collected during the observation period were pooled prior to analyses. Fiber fractions (neutral detergent fiber, NDF; acid detergent fiber, ADF; and acid detergent lignin, ADL) were determined following the methods reported by Van Soest et al. [18], after treatment with thermostable alpha-amylase in a sodium sulfite solution. AOAC methods [19] were used to determine crude protein, crude fat, and ash.

Fatty acid methyl esters (FAME) were prepared by combining extraction and methylation in a 1-step procedure, using chloroform to extract lipid and 2% (v/v) sulfuric acid in methanol for methylation [20–21]. Nonadecanoic acid (19:0) was the internal standard. Individual FAME were separated and quantified as later reported for meat fatty acids. Individual fatty acids were expressed as g/100 g of total fatty acids.

Tocopherols in 200 mg of freeze-dried feeds were determined as explained later for meat samples.

The procedure described by Bolletta et al. [22] was used for total phenols and tannin quantification. Briefly, 200 mg of finely ground forage dissolved in a 70:30 (v/v) acetone-water solution underwent sonication in a water bath at 4°C for 15 min. Samples were agitated under attenuated light in a rotary shaker for 2 hours. After centrifugation, the upper phase, representing the phenolic extract, was recovered, and treated with Folin-Ciocalteu reagent (1N) and sodium carbonate 20% (w/v). Total phenolic compounds were quantified by a double-beam UV/VIS spectrophotometer (UV-2550; Shimadzu Corporation, Milan, Italy). Non-tannin phenols were quantified using the same procedure, after removing tannin by means of insoluble polyvinylpolypyrrolidone (PVPP). Tannins were calculated as the difference between total phenols and total non-tannin phenols. Quantification of phenolic compounds was achieved using standard solutions of tannic acid (TA) and data were expressed as g TA equivalents/kg dry matter.

### Meat analyses

For the fatty acid profile, intramuscular fat was extracted from 10 grams of muscle according to Folch et al. [23] using a mixture of chloroform and methanol (2:1, v/v). FAMEs were prepared by base-catalysed transesterification [24] using sodium methoxide in methanol (0.5 N) and hexane containing nonadecanoic acid (C19:0; 72332, Merck KGaA, Darmstadt, Germany) as an internal standard. Gas chromatographic separation was performed in a 100-m high-polar fused silica capillary column (SP – 2560 fused silica, Supelco, Bellafonte, PA, 100 m  × 0.25 mm i.d.; film thickness 0.25 μm) installed on a Thermo Finnigan Trace GC Ultra with a flame ionization detector (FID; TRACE GC Ultra, ThermoQuest, Milan, Italy). Helium (1 mL/min constant flow) was the carrier gas. Total FAME profile in a 1 μL sample volume (split ratio 1:80) was determined using the conditions reported by Bolletta et al. [22]. Specifically, an initial temperature of 50 °C for 1 min, then the following ramps were programmed: 10 °C/min increase until 120 °C for 1 min; 5 °C/min increase until 180 °C for 18 min; 2 °C/min increase until 200 °C for 15 min; 2 °C/min increase until 230 °C for 19 min. Constant temperatures for injector and detector were 270 °C and 300 °C, respectively. Individual FAMEs were identified by comparing the retention time with those of commercially available FAME standard mixtures (GLC-463, Nu-Chek Prep Inc., Elysian, MN, USA; Larodan Fine Chemicals, Malmo, Sweden) and by comparison with chromatograms available in the literature [25]. Fatty acid quantification was performed based on the ratio between the peak area of each fatty acid and that of the internal standard, and fatty acids were expressed as mg/100 g of meat and as g/100 g of total fatty acids.

Tocopherols in meat were determined according to Bolletta et al. [22] with modifications. After homogenization with a butylated hydroxytoluene solution (0.06% in ethanol), 2 grams of meat were saponified by adding 60% KOH and incubating at 70 °C for 30 minutes in the dark. The sample was then centrifuged (7600 × g), and the supernatant was extracted three times using a hexane/ethyl acetate mixture (9:1, v/v). The solvent was evaporated under a nitrogen stream, and the remaining residue was dissolved in methanol. For tocopherols quantification, 10 μL of sample was injected into a UHPLC system (Nexera, Shimadzu Corporation, Kyoto, Japan) equipped with a C18 reversed-phase column (Zorbax ODS; 25 cm × 4.6 mm, 5 μm; Supelco, Bellefonte, PA, USA). The mobile phase consisted of methanol at a flow rate of 1.3  mL/min. Tocopherols were detected using a spectrofluorometric detector with an excitation wavelength of 295 nm and an emission wavelength of 330 nm. Analyte identification was performed by comparing retention times with those of pure standards. Quantification of tocopherols was performed using external calibration curves prepared in ethanol with increasing amounts of pure α-tocopherol and γ-tocopherol (T3251 and T1782, Sigma-Aldrich, Bornem, Belgium).

Antioxidant capacity of meat was estimated using ferric reducing antioxidant power (FRAP) and trolox-equivalent antioxidant capacity (TEAC) assays. A meat extract was prepared by mixing 2 grams of meat and 10 mL of distilled water in a 50 mL Falcon tube. After 1-minute homogenization in ice bath and filtration (Whatman No.1 paper filter), 50 µL and 20 µL of the filtered sample were used for FRAP and TEAC determination, respectively. FRAP assay was performed according to Benzie and Strain [26]. Briefly, the extract was added to a solution 50:5:5:6 of pH 3.6 acetate buffer (300 mM sodium acetate trihydrate in 1.6% acetic acid), 0.01 M TPTZ [2,4,6-tris(2-pyridyl)-s-triazine] in 0.04 M hydrochloric acid, 0.02 M ferric chloride hexahydrate and distilled water. Dark incubation (1 h at 37 °C) was immediately followed by absorbance reading at 593 nm using a double beam UV/VIS spectrophotometer (UV-1601, Shimadzu Corporation, Milan, Italy). FRAP was expressed as mmol of Fe(II) equivalent per gram of meat based on a calibration curve built using ferrous sulphate. A modified procedure of Re et al. [27] was used for TEAC assay. Briefly, an activated radical solution was prepared by incubating in the dark at room temperature for 14 hours a mix (1:1 v/v) of 4.9 mM potassium persulfate and 14 mM ABTS (2,2-azinobis-3-ethylbenzothiazoline-6-sulfonic acid). Two mL of activated radical solution, diluted until the absorbance at 734 nm reached 0.75 ± 0.02, was added to the meat extract. Immediately after 60 min dark-incubation at 30 °C, the absorbance at 734 nm was read. The reduction of absorbance was compared to a blank, and to an external calibration curve prepared using 2.5 mM Trolox solution. Results were expressed as mmol of TEAC per gram of meat.

Myoglobin determination was performed following the procedure described by Monahan et al. [28]. Two grams of finely minced meat were added with 25 mL distilled water and homogenized in ice-bath. Then, the sample was centrifuged at 6000 × *g* for 15 minutes at 4 °C and filtered (Whatman No. 1 filter). Finally, the absorbance recorded at 503 nm, 525 nm, 557 nm, 582 nm, and 730 nm by a UV/VIS spectrophotometer (UV-1601, Shimadzu Corporation, Milan, Italy) was retained. Krzywicki’s equation [29] was used to calculate myoglobin concentration (mg/g of meat).

Color and lipid oxidation development over time was monitored on three slices (2 cm-thick) knife-cut from each sample and placed into polystyrene trays. After wrapping with oxygen-permeable PVC film (avoiding contact between the meat surface and the PVC film), the meat was stored at 4 °C from 0 to 7 days. One slice was used after 2 h blooming (d0) to determine color parameters in the CIELAB space (lightness, L*; redness, a*; yellowness, b*; chroma, C*; hue angle, H*) using a Minolta CM-2022 spectrophotometer (Minolta Co., Ltd. Osaka, Japan) set as follows: d/8° geometry; illuminant A; 10° standard observer. The other two slices were used for color parameter determination after 3 days (d3) and 7 days (d7) of refrigerated storage, respectively. Three readings per sample were made and the average value was retained for each parameter. The accumulation of metmyoglobin on the surface of the slice was estimated by the (K/S)572÷(K/S)525 ratio [30].

The same samples intended for color determination were also used for 2-thiobarbituric acid reactive substances (TBARS) analysis to assess lipid oxidation [31]. Briefly, 2.5 grams of muscle were first homogenized (IKA, T-18 basic UltraTurrax, KA-Werke GmbH & Co.KG, Staufen, Germany) with 12.5 mL of distilled water and then added with 12.5 mL of 10% (w/v) trichloroacetic acid to precipitate the proteins. Then, the sample was filtered (Whatman No. 1 filter paper) and 4 mL of filtrate were transferred to a pyrex tube to be treated with 1 mL of 0.06 M aqueous thiobarbituric acid and incubated in a water bath at 80 °C for 90 min. Finally, absorbance at 532 nm was read on a Shimadzu UV–vis spectrophotometer (UV-1601; Shimadzu Corporation, Milan, Italy). Solutions of known concentration of TEP (1,1,3,3-tetraethoxypropane) were used to build a calibration curve for TBARS quantification. TBARS were expressed as mg of malonaldehyde/kg of meat.

### Indexes calculation

Data on meat fatty acids were used to calculate health indexes related to the capability of meat lipid to favor the formation of atherogenic plaques (AI) and thrombotic coagula (TI) in the blood or to increase the cholesterol level in the bloodstream (h/H). Specifically, AI and TI were calculated using the equations of Ulbricht and Southgate [32]:


AI = (12:0 + 4 * 14:0 + 16:0)/ (MUFA + n−6 PUFA + n−3 PUFA)

TI = (14:0 + 16:0 + 18:0)/ [(0.5 * MUFA) + (0.5 * n−6 PUFA) + (3 * n−3 PUFA) + (n−3/n−6 PUFA)]

The hypocholesterolemic to hypercholesterolemic fatty acids ratio (h/H) was calculated according to Mierlita [33] as follows:


h/H = (18:1 c9 + PUFA)/ (12:0 + 14:0 + 16:0)

The concentration of highly peroxidable fatty acids (HP-PUFA) was calculated as the sum of fatty acids with 2 or more unsaturations, while the peroxidability index (PI) was calculated as reported by Menci et al. [34]:


PI = (Σ dienoic) + (Σ trienoic *2) + (Σ tetraenoic *3) + (Σ pentaenoic *4) + (Σ hesaenoic *5)

### Statistical analysis

The effect of the feeding season (WIN, SPR or SMM) on animal performance, meat fatty acids, antioxidant capacity, and myoglobin content was analyzed by a mixed model with diet as fixed factor and individual animal as random factor. Data on meat color parameters and lipid oxidation were analyzed using a mixed model to test the effect of feeding season (WIN, SPR, SMM) and days of refrigerated storage (d0, d3, and d7 days) as fixed factors, and their interactive effect. Individual animal was considered the random effect. As farming time significantly differed between groups, it was used as a covariate to control for its potential confounding effect and to better isolate the effects attributable to feeding season. Differences between pairwise comparisons of the means were assessed by Tukey’s adjustment. Significance was declared when P ≤ 0.05, while trend towards significance were considered for 0.05 < P ≤ 0.10.

All meat quality data (for TBARS, colorimetric parameters, and metmyoglobin percentage, the mean of the three measurements collected at different refrigeration time points was used) and farming time also underwent a Principal Component Analysis (PCA, Minitab) for unveiling potential relationships between the diet consumed in the last period and meat quality, and a multivariate linear discriminant analysis (SPSS, version 18.0.0, 2009). As for the latter, a stepwise variable selection procedure was applied to identify the subset of variables that most effectively differentiated the feeding seasons, following the methodology described by Valenti et al. [35]. Specifically, variables with an F-value lower than 0.10 at the final step of the selection process were included in a canonical discriminant analysis. This analysis generates a canonical function (CAN) composed of the most informative quantitative variables, summarizing the overall variance and optimizing group separation. The model’s discriminant power was assessed via Wilks’ Lambda significance test. Its classification performance was determined using leave-one-out cross-validation, in which each animal was classified using canonical functions derived from all other individuals in the dataset. The higher the classification accuracy, the greater the model’s ability to correctly assign individuals to their original group.

## Results

Animals in the WIN group had a shorter time on farm than SPR and SMM animals (P = 0.001), while all other productive traits did not show significant difference (Table 2).

**Table 2:** Performance of the 38 monitored grass-fed Angus steers.

	Feeding Season^1^			SEM^*2*^	P-value^*3*^
	WIN	SPR	SMM		
**Number of animals**	13	13	12		
**Age at arrival (moths)**	12.51	11.59	11.77	0.23	0.227
**Body weight ad arrival (kg)**	316.24	282.00	314.07	8.11	0.147
**Farming time (months)**	12.05^b^	14.10^a^	13.69^a^	0.26	0.001
**Final live weight (kg)**	563.31	583.77	586.18	10.92	0.651
**Average daily gain (g)**	670.74	705.80	657.49	14.56	0.389
**Hot carcass weight (kg)**	313.64	322.65	326.34	7.07	0.764
**Cold carcass weight (kg)**	305.89	316.38	317.33	7.03	0.768
**Carcass yield (%)**	54.14	54.06	54.17	0.46	0.996
**Cold carcass pH**	5.64	5.59	5.48	0.10	0.875

^1^Feeding season: WIN = Hay-based diet, with hay ≥ 80% total ingestion); SPR = Spring Pasture-based diet, with pasture ≥ 80% total ingestion diet; SMM = Summer Pasture-based diet, with pasture ≥ 80% total ingestion diet).

^2^SEM: Standard error of mean.

^3^P-value of the effect of the feeding season; ^a-b^ Means in the same row with different superscripts are significantly different (P ≤ 0.05).

The diet ingested by the animals in the last three months before slaughter moderately affected meat fatty acids concentrations (Table 3). Fat percentage did not differ (P > 0.05) between groups. Differences (P ≤ 0.05) were observed only for individual PUFA. Specifically, 18:2 *t*9*t*12 concen*t*ration was greater in the WIN meat in comparison to SMM meat, 18:3 n-6 was greater in the meat of WIN than the two other groups, and 20:5 n-3 was lower in WIN than SMM meat. Moreover, meat from WIN animals tended (P ≤ 0.10) to show lower concentration of 20:2 n-6 than the two other groups, and lower concentration of 22:5 n-6 than SPR meat. Similarly, HP-PUFA and PI tended to be lower (P ≤ 0.10) in WIN meat than SPR meat. The meat from SPR and SMM groups showed greater content of α-tocopherol (P ≤ 0.05) than the WIN group, whereas γ-tocopherol was greater in SPR meat than SMM meat. The feeding season did not affect the relative proportion of individual fatty acids (S1 table).

**Table 3:** Effect of diet ingested in the three months before slaughter on meat fat percentage and fatty acids.

	Feeding Season^*1*^			SEM^*2*^	P-value^*3*^
	WIN	SPR	SMM		
**Fat %**	2.68	2.68	2.57	0.194	0.848
**Fatty Acids (mg/100g meat)**					
**12:0**	1.47	2.16	2.62	0.39	0.495
**12:1 *c*9**	0.44	0.62	0.60	0.09	0.641
**14:0**	31.26	45.86	40.43	6.77	0.836
**14:1 *c*9**	13.68	18.16	13.12	1.62	0.387
**15:0 *iso***	7.11	6.70	7.64	0.85	0.909
**15:0 *anteiso***	8.59	8.35	9.27	1.05	0.938
**15:0**	18.09	18.52	17.44	2.15	0.918
**16:0**	632.52	655.86	595.32	47.20	0.878
**16:1 *c*9**	77.32	83.23	74.47	5.98	0.838
**17:0 *iso***	11.59	12.05	11.73	0.82	0.491
**17:0 *anteiso***	20.16	19.97	19.94	1.01	0.796
**17:0**	39.07	39.48	37.98	1.84	0.786
**17:1 *c*9**	21.97	22.84	22.78	3.84	0.676
**18:0**	374.20	319.53	335.62	1.67	0.720
**18:1 *t*6-7–8**	1.17	1.56	1.28	28.00	0.713
**18:1 *t*9**	3.85	4.25	3.71	0.19	0.860
**18:1 *t*10**	2.71	2.05	2.33	0.41	0.526
**18:1 *t*11**	40.65	34.58	41.42	0.24	0.777
**18:1 *c*6**	4.29	3.96	4.09	4.22	0.961
**18:1 *c*9**	782.24	781.86	729.02	0.48	0.883
**18:1 *c*11**	47.29	41.33	53.18	53.30	0.634
**18:1 *c*12**	6.19	3.65	6.88	4.92	0.184
**18:1 *c*13**	6.58	9.13	5.96	0.75	0.232
**18:2 *t*9*t*12**	1.61^a^	0.97^ab^	0.223^b^	0.11	0.039
**18:2 *c*9*t*11**	10.29	9.80	10.23	1.03	0.873
**18:2 n-6**	87.91	109.53	99.61	6.07	0.347
**18:3 n-6**	0.54^b^	0.98^a^	0.69^a^	0.06	0.006
**18:3 n-3**	34.54	39.53	35.45	2.14	0.604
**20:0**	3.64	2.78	7.27	1.41	0.406
**20:2 n-6**	1.12^b^	1.76^a^	1.64^a^	0.12	0.071
**20:3 n-6**	7.30	9.49	8.26	0.43	0.106
**20:3 n-3**	1.07	1.38	1.27	0.09	0.347
**20:4 n-6**	1.07	1.38	1.27	2.25	0.170
**20:5 n-3**	23.57^b^	33.07^a^	24.89^ab^	0.46	0.022
**22:0**	0.74	0.94	0.58	0.09	0.254
**22:4 n-6**	1.62	2.19	4.03	0.65	0.306
**22:5 n-6**	0.19	1.04	0.30	0.17	0.080
**22:5 n-3**	12.72	14.44	13.49	0.81	0.692
**22:6 n-3**	1.25	1.48	1.71	0.17	0.573
**SFA** ^**4**^	1069.92	1086.74	1036.44	81.60	0.970
**MUFA** ^**5**^	1008.37	1043.31	989.84	70.30	0.945
**PUFA** ^**6**^	189.59	243.74	216.42	10.80	0.113
**OBCFA** ^**7**^	104.62	108.96	107.36	10.80	0.789
***t*10/*t*11 18:1**	0.09	0.07	0.06	0.09	0.496
**PUFA n-6**	122.25	158.06	139.44	8.52	0.225
**PUFA n-3**	57.05	67.17	59.98	2.96	0.359
**PUFA n-6/n-3**	2.24	2.38	2.59	0.18	0.735
**AI** ^**8**^	0.70	0.72	0.68	0.025	0.835
**TI** ^**9**^	1.39	1.26	1.28	0.04	0.437
**HP-PUFA** ^**10**^	90.27^b^	113.94^a^	98.16^ab^	4.60	0.093
**PI** ^**11**^	349.56^b^	446.98^a^	398.02^ab^	18.70	0.086
**h/H** ^**12**^	1.49	1.48	1.55	0.05	0.613
**Vitamins and cholesterol (mg/100 g meat)**					
**α-tochopherol**	0.95^**b**^	1.47^**a**^	1.79^**a**^	1.12	0.005
**γ-tochopherol**	0.17^**b**^	0.19^**a**^	0.18^**ab**^	0.03	0.040

^1^Feeding season: WIN = Hay-based diet. with hay ≥ 80% total ingestion); SPR = Spring Pasture-based diet, with pasture ≥ 80% total ingestion diet; SMM = Summer Pasture-based diet, with pasture ≥ 80% total ingestion diet)

^2^SEM: Standard error of mean

^3^P-value of the effect of feeding season; ^a-b^ Means in the same row with different superscripts significantly differ (P ≤ 0.05) or tend to differ (0.05 < P ≤0.10)

^4^SFA: Sum of Saturated Fatty Acids

^5^MUFA: Sum of Monounsaturated Fatty Acids

^6^PUFA: Sum of Polyunsaturated Fatty Acids

^7^OBCFA: Sum of Odd and Brunched Chain Fatty Acids

^8^AI: Atherogenic index, (12:0 + 4 * 14:0 + 16:0)/ (MUFA + PUFA n-6 + n-3 PUFA)

^9^Thrombogenic index, (14:0 + 16:0 + 18:0)/ [(0.5 * MUFA) + (0.5 * n-6 PUFA) + (3 * n-3 PUFA) + (n-6/n-3 PUFA)]

^10^HP-PUFA: Highly peroxidizable polyunsaturated fatty acids calculated as the sum of PUFA with three or more double bonds;

^11^PI: Peroxidability index, (∑Dienoic fatty acids) + (∑Trienoic fatty acids*2) + (∑Tetraenoic fatty acids*3) + (∑Pentaenoic fatty acids*4) + (∑Hexaenoic fatty acids*5)

^12^h/H: hypocholesterolemic to hypercholesterolemic fatty acids ratio (18:1 *c*9 + PUFA)/ (12:0 + 14:0 + 16:0)

Table 4 reports the effect of diet ingested in the last three months before slaughtering on myoglobin content and antioxidant capacity of meat. Myoglobin content was lower in WIN meat than in SPR and SMM meat (P ≤ 0.05), whereas no difference (P > 0.05) was observed for metmyoglobin. FRAP did not differ between groups (P > 0.05), whereas TEAC tended to be greater (P ≤ 0.10) in SMM meat than WIN meat.

**Table 4:** Effect of diet ingested in the three months before slaughteron meat myoglobin content and antioxidant capacity.

	Feeding Season^*1*^			SEM^*2*^	p-value^*3*^
	WIN	SPR	SMM		
**Myoglobin (mg/g meat)**	4.1^b^	5.1^a^	5.0^a^	0.17	0.011
**Antioxidant capacity**					
**FRAP** ^**4**^	353.4	449.7	380.1	21.70	0.167
**TEAC** ^**5**^	1.29^b^	1.39^ab^	1.55^a^	0.05	0.085

^1^Feeding season: WIN = Hay-based diet, with hay ≥ 80% total ingestion); SPR = Spring Pasture-based diet, with pasture ≥ 80% total ingestion diet; SMM = Summer Pasture-based diet, with pasture ≥ 80% total ingestion diet)

^2^SEM: Standard error of mean

^3^P-value of the effect of the feeding season; ^a-b^ Means in the same row with different superscripts significantly differ (P ≤ 0.05) or tend to differ (0.05 < P ≤0.10)

^4^FRAP: Ferric Reducing Antioxidant Power expressed as mmol of Fe(II) equivalent per gram of meat

^5^TEAC: Trolox Equivalent Antioxidant Capacity expressed as mmol of TEAC per gram of meat

**Fig 1:**
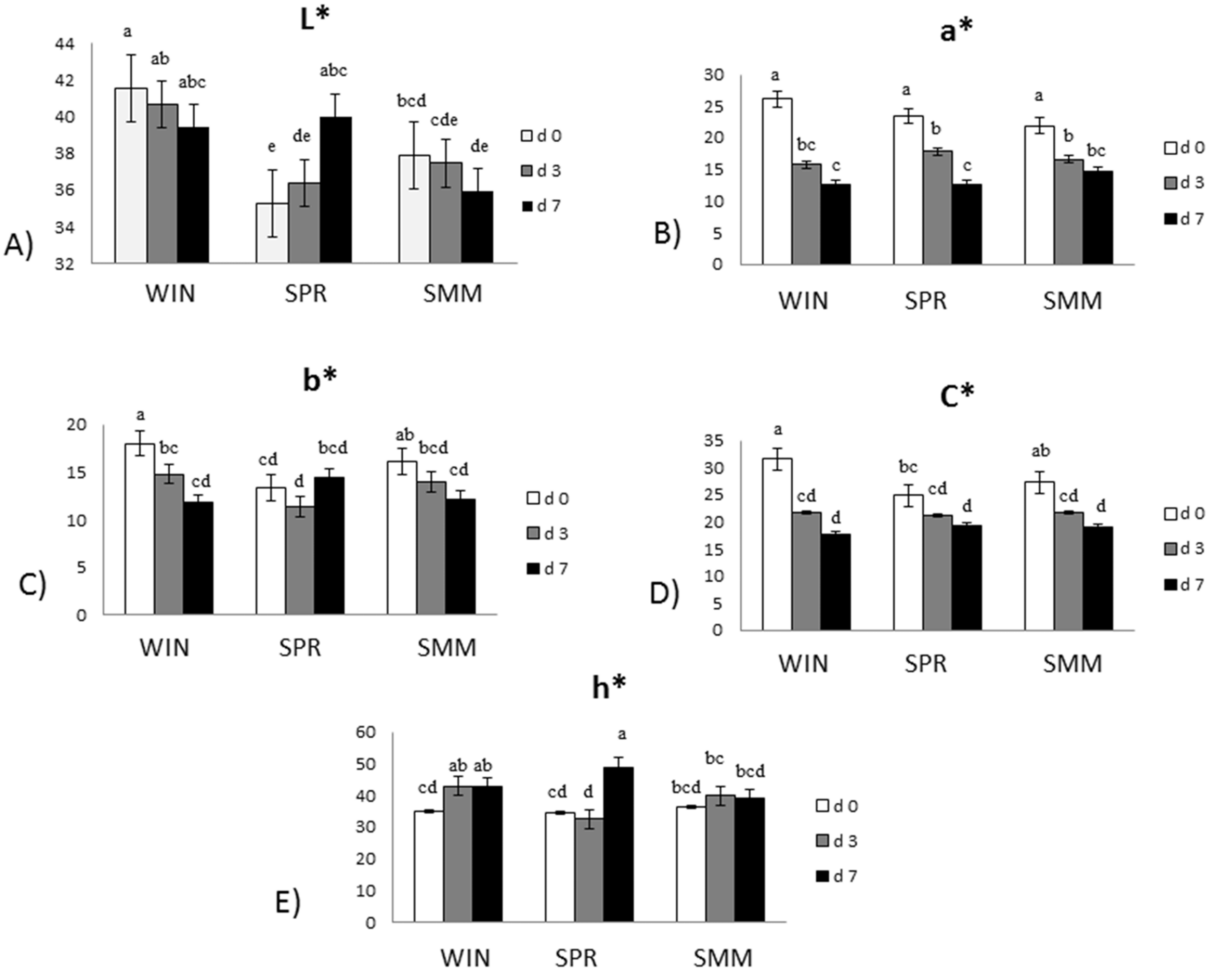
Interactive effect of feeding season (WIN, SPR or SMM) and time (d0, d3 and d7) of refrigerated storage on meat color parameters evolution. WIN = Hay-based diet, with hay ≥ 80% total ingestion); SPR = Spring Pasture-based diet, with pasture ≥ 80% total ingestion diet; SMM = Summer Pasture-based diet, with pasture ≥ 80% total ingestion diet). ^a-d^ Different superscripts indicate significant difference (P ≤ 0.05).

Fig 1 reports the interactive effect of the seasonal diet and refrigerated storage on meat color parameters. An interactive effect was reported for all the investigated parameters. Lightness (Fig 1-a, P < 0.001) showed different evolution over time in the three groups. Specifically, WIN meat had the greatest value at d0 in comparison with SPR and SMM. Moreover, WIN meat showed comparable L* at the 3 times of storage (d0, d3, and d7). Similarly, in SMM meat L* was comparable over time. Conversely, an increasing trend was reported for the L* of SPR meat, which showed the lowest value at d0 and increased at d7 as compared to d0 and d3. Redness (Fig 1-b, P = 0.005) was greater at d0 despite the feeding season. However, progressive degradation of a* from d0 to d3 to d7 was observed only for SPR meat, whereas a* degraded faster in WIN and SMM meat, with a minimum level recorded already at d3. Yellowness (Fig 1-c, P < 0.001) developed differently in the meat of each dietary group. WIN and SMM meat started from greater value than SPR meat. However, b* decreased already at d3 to remain stable at d7 in WIN meat, whereas SMM meat showed lower values at d7 than d0. SPR meat showed comparable b* values over storage time. C (Fig 1-d, P < 0.001) showed similar evolution over time for WIN and SMM meat, with higher values recorded at d0 than d3 and d7. Conversely, in SPR meat, C lowered only after 7 days of storage in comparison with d0. Hue (Fig 1-e, P < 0.001) progressed differently based on dietary groups. WIN meat showed greater H values at d3 and d7 than at d0. SPR meat showed a greater value at d7 in comparison with d0 and d3. Hue did not differ over time for SMM meat.

**Fig 2:**
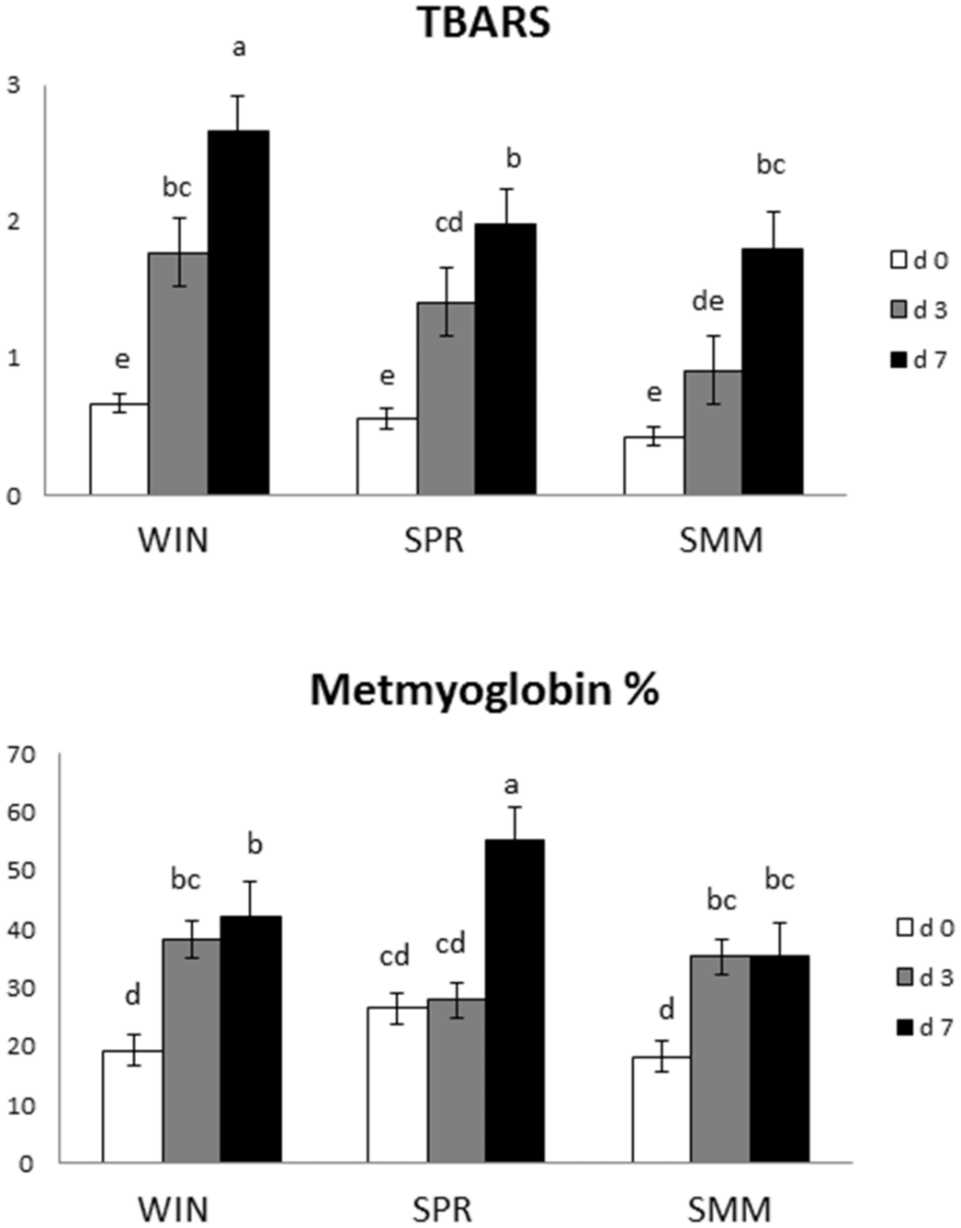
Interactive effect of feeding season (WIN, SPR or SMM) and time (d0, d3 and d7) on meat lipid oxidation (TBARS). WIN = Hay-based diet, with hay ≥ 80% total ingestion); SPR = Spring Pasture-based diet, with pasture ≥ 80% total ingestion diet; SMM = Summer Pasture-based diet, with pasture ≥ 80% total ingestion diet). ^a-d^ Different superscripts indicate significant difference (P ≤ 0.05).

Fig 2 describes the evolution of meat lipid oxidation and metmyoglobin formation on the meat surface as affected by the interaction between the diet consumed during the last three months before slaughter and time of refrigerated storage (P = 0.035 and P < 0.001 for TBARS and Metmyoglobin percentage, respectively). All the samples showed similar TBARS concentration at d0, but TBARS progressively increased in SPR and WIN meat moving from d0 to d3 to d7. Also, WIN meat showed the greatest TBARS concentration at d7. Conversely, SMM meat showed greater TBARS only at d7 than d0 and d3. Metmyoglobin percentage was comparable between groups at d0 and d3, while the highest value was observed on the surface of SPR meat at d7. Moreover, metmyoglobin showed different increasing trends: it reached maximum percentage already after 3 days of refrigerated storage in the meat of WIN and SMM group, whereas for the SPR meat it increased only on the last day of storage as compared to d0 and d3.

The analysis of the principal components was unable to clearly separate the meat based on the feeding season (S1 Fig). Conversely, the stepwise procedure of the discriminant analysis allowed to retain seven quality parameters from the entire dataset (17:1 *c*9, 18:1 *t*10, 22:0, 22:5 n-6, b*, TBARS and metmyoglobin %) in addition to the farming time (Table 5) that linearly combined into two canonical discriminant functions (CAN) explained the total variance of the dataset.

**Table 5:** Standardized coefficients of canonical functions (CAN) and explained variance.

	CAN1	CAN2
**17:1 c9**	1.369	−1.276
**18:1 *t*10**	−1.700	1.125
**22:0**	−0.955	0.470
**22:5 n-6**	1.054	0.150
b*	−1.083	0.387
**TBARS** ^**1**^	−0.123	1.303
**Metmyoiglobin %**	1.207	0.828
**Farming time**	0.580	0.236
**Explained variance (%)**	69.8	30.2

^1^Thiobarbituric acid reactive substance

**Fig 3:**
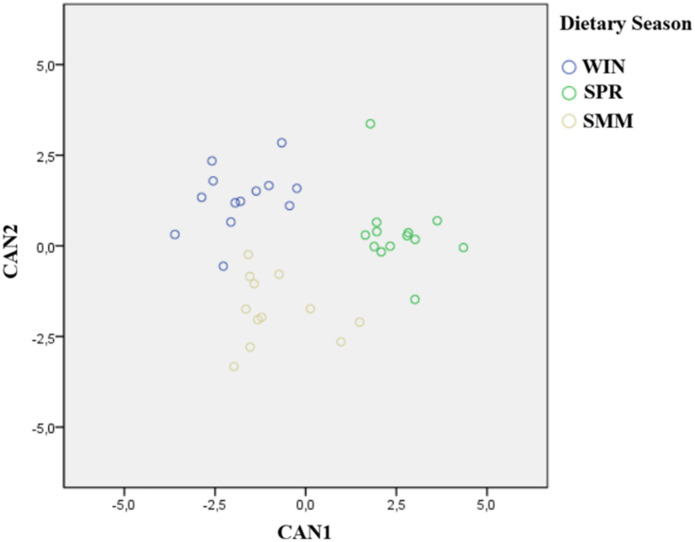
Discrimination of the feeding season obtained by plotting the canonical functions (CAN1 and CAN2) obtained from the whole dataset of meat quality parameters after the stepwise procedure. WIN = Hay-based diet, with hay ≥ 80% total ingestion); SPR = Spring Pasture-based diet, with pasture ≥ 80% total ingestion diet; SMM = Summer Pasture-based diet, with pasture ≥ 80% total ingestion diet).

The scatterplot (Fig 3) represents the distribution of the meat samples in the multivariate space, with CAN1 (69.8% of the total variance) and CAN2 (30.2% of the total variance) as the X and Y axes, respectively. CAN 1 allowed discrimination between SMM and the two other groups. CAN2 partially separated the samples from the three groups. After the cross-validation, 89.5% of cases were correctly assigned to the feeding group. As for the wrongly assigned samples: 1 SPR meat sample was misclassified and assigned to WIN; 1 SMM meat sample was assigned to SPR and 1 to the WIN group; 1 WIN meat sample was assigned to SMM. Table 5 reports the standardized coefficients of the variables describing the total canonical structure. The absolute value of the standardized coefficients of each variable represents a measure of the relative contribution that the variable itself provides to the discriminating capacity of the whole multivariate function. Therefore, a greater value means a greater discriminating contribution. Among the variables retained after the stepwise procedure, 17:1 *c*9, 18:1 *t*10 and metmyoglobin% greatly influenced the discriminant power of both the CAN1 and CAN2, while farming time showed the lowest coefficients for both functions. Among the other variables, b*, 22:0 and 22:5 n-6 contributed to the discriminant power of CAN1, whereas TBARS mostly accounted in the CAN2.

## Discussion

The socio-cultural context represents one of the factors that may affect consumer purchasing choices. In turn, the consumer, with their choices, can influence the way the industry operates, including the food industry. The ongoing climate change is moving consumer preferences towards food products obtained through environmentally friendly systems and, in the animal field, respectful of animal welfare. Grass-fed meat perfectly meets these demands, and for these reasons, its market has constantly grown in recent years [36]. The demand for sustainable products has not changed those related to quality. Rather, the latter has been reinforced in light of the increasing consumer awareness of the role of nutrition in human health. Therefore, for grass-fed farmers it is pivotal, in addition to achieving acceptable growth rates, to ensure that the meat consistently maintains a high chemical-nutritional and sensory quality. One of the biggest challenges in guaranteeing this aspect could lie in the variability of the diet ingested by the animals during the rearing phase, particularly in the last months before slaughter [37].

The present study assessed the meat quality parameters of Angus steers raised on the same farm according to a grass-fed system and slaughtered at the same target weight in different periods of the year after receiving different forage diets in the last months before slaughter.

In a grass-fed system, animal diet consists of forages as the only feed except for the nursing phase. Unlike conventional systems, where animals are fed concentrates and have limited mobility, grass-fed animals receive a diet with lower energy density, and they spend some of this energy procuring feed by moving. As a result, animal growth rates are slower. Previous studies on Angus cattle raised in a grass-fed system reported daily weight gains of 0.7 kg compared to more than 1 kg/day for animals of the same breed fed concentrates [38]. In our conditions, the average daily gain observed since the arrival at the farm was 0.6 kg/day. Comparable data were also obtained by Klopatek et al. [39] with the same breed in similar conditions. In the present study, WIN animals showed a shorter on-farm period (about two months) compared with the SPR and SMM groups, although other productive performances did not differ. Indeed, despite *a posteriori* selection of the 38 animals aimed at minimizing initial differences between groups (in age and body weight), SPR animals had numerically lower initial body weights and likely required a longer period to reach the target weight. Considering the survey nature of the study, such discrepancies may be expected and reflect normal variability under practical farm conditions rather than controlled experimental differences.

Fatty acid composition is one of the most important meat qualitative traits related to human health [40] and meat oxidation [41]. Overall, ruminant meat is considered unhealthy due to a high concentration of saturated fatty acids, and an n-6/n-3 PUFA ratio higher than the recommended human intake limit of 4. However, it should be emphasized that fat composition largely varies depending on the animal diet [42]. The role of dietary green herbage in increasing n-3 PUFA levels in animal products is widely recognized [43]. Thanks to the abundance of dietary green herbage, grass-fed meat shows levels of unsaturated and n-3 fatty acids closer to the recommended values [6,40]. This is why a finishing period on grazing can be used as an effective strategy to partially reduce the n-6/n-3 PUFA ratio in grain-fed cattle meat. In our study, the n-6/n-3 PUFA ratio was always below 4, and showed no differences between seasons, as well as the other health indexes (AI, TI and h/H). Similar results to those observed in this study have been reported for meats obtained in grass-fed or forage-based systems [39,43,44].

In the context of forage-based feeding systems, Krusinski et al. [45] highlighted seasonal plant variations (i.e., plant type, phenological stage, leaf-to-stem ratio, etc.) as important factors capable of affecting the fatty acid profile of meat. These authors reported that 18:3 n-3 (the main n-3 fatty acid in forages) is highest during spring when plants are young and leaves predominate over stems, but progressively decreases throughout the seasons. Conversely, 18:2 n-6 (the main n-6 fatty acid in forages), 18:1 *c*9, and 16:0 follow an opposite trend. Accordingly, previous studies, though lacking specific details regarding dietary composition, indicate that meat from animals in grass-fed or forage-based systems contains higher quantities of total PUFAs and n-3 and n-6 fatty acids when slaughtered in the spring or summer compared to the winter [16,46]. In our trial, the variation in dietary fatty acids was consistent with the literature. Nevertheless, the fatty acid profile of the meat exhibited minimal differences between seasons. None of the sums of fatty acids showed significant differences. Only 18:3 n-6, 20:5 n-3, and 22:5 n-6 were higher in the SPR meat than in the WIN meat. Although these findings partially align with dietary fatty acids, larger differences could have been expected considering the higher percentage of PUFA in the SPR and SMM diets compared to the WIN diet and previous literature [16]. Besides the muscle selected for evaluation, other factors may have contributed to masking potential differences. It is known that the lower the intramuscular fat content the greater the contribution of PUFA in defining the fatty acid profile of meat [47]. Furthermore, although it is recognized that the diet ingested by the animals in the last pre-slaughter period may affect meat lipid profile, the latter represents the results of fatty acid accumulation in adipose tissues throughout the entire farming period. In our study, both hay and green herbage were present across all feeding seasons, which may have contributed to the lack of remarkable differences, or the duration of the finishing period may not have been long enough to reveal potential differences in these conditions. Slight differences in fatty acids composition were obtained also by Pestana et al. [14] evaluating meat quality in a semi-extensive system.

Vaccenic acid (18:1 t*1*1) predominated among *trans*- monounsaturated fatty acids, and the *t*10/*t*11 18:1 ratio remained below 1 across the feeding seasons. Similarly, the ratio between non-*t*11 18:1 isomers and 18:1 *t*11 was always below 1. These observations agree with the increase in vaccenic acid levels in ruminant products at the expense of other *trans* 18:1 isomers when their diet is rich in forages [48–49]. These outcomes are desirable, as the health effects of trans fatty acids can differ depending on their origin. Indeed, Oteng and Enser [50] highlighted that industrial trans fatty acids can promote inflammation, fat storage in the liver, and elevate plasma low-density lipoproteins and cholesterol, whereas ruminant *trans* 18:1 fatty acids increase cholesterol associated with high-density plasma lipoproteins and alleviate metabolic disorders. This difference seems to stem from both the distribution and the structural nature of the individual *trans* 18:1 isomers. In particular, industrial *trans* fatty acids differ structurally from those naturally occurring in ruminant fat, as the double bonds are located at different positions along the carbon chain. Industrial products typically contain a more homogeneous mix of positional isomers which are present at comparable proportions. In contrast, ruminant-derived *trans* fatty acids originate from biohydrogenation processes in the rumen, resulting in the predominance of specific positional isomers that are subsequently transferred to meat, as observed for the 18:1 *t*11 in our study.

Lipid oxidation is one of the primary processes responsible for the qualitative degradation of meat, significantly contributing to a reduced shelf life [51]. Unsaturated fatty acids, particularly PUFA, are more susceptible to oxidative processes than SFA [52]. During storage, they initially undergo oxidation, forming peroxides, followed by the generation of secondary oxidation products [53]. These compounds negatively affect sensory properties, such as the smell, taste, and color of the meat, but can also exert toxic effects, contributing to cellular damage as in the case of malondialdehyde [54]. Therefore, while it is nutritionally beneficial to increase PUFA content in meat, it is equally crucial to prevent their oxidation. In the present study, meat samples were assessed for seasonal variation in oxidative stability by measuring the content of fatty acids prone to oxidation and the evolution of thiobarbituric acid reactive substances at different refrigeration intervals. The TBARS concentrations observed in the meat samples were within the range reported for meat obtained under comparable conditions. However, direct comparisons among studies remain difficult. In particular, TBARS values at d0 were similar to those reported by Hughes et al. [55], although a different muscle was analyzed in that study and the effect of refrigerated storage was not assessed. Moreover, TBARS values in the present study were higher than those reported by Yang et al. [56], where the meat was vacuum-aged for twice the ageing period. In our study, despite minor differences in fatty acid profile among the three groups, WIN meat tended to show a lower concentration of highly peroxidizable PUFAs and a reduced peroxidability index compared to SPR group. However, WIN meat exhibited greater lipid oxidation products already after three days of refrigerated storage compared to meat from the SMM group and the greatest content than the other groups at the end of the 7-day monitoring period. This outcome aligns with a general trend of increasing antioxidant capacity (FRAP) observed from WIN to SPR and SMM meats, likely attributable to the lower pre-slaughter ingestion of antioxidants by WIN animals. In the literature, tocopherols are widely recognized as potent natural antioxidants improving lipid oxidative stability and their content in the meat increases along with dietary content [57–58]. Natalello et al. [59] found lower TBARS values in meat from lambs fed a dietary source rich in vitamin E as compared to the control meat despite a greater content of high peroxidable fatty acids. Similar results have been observed by Duarte et al. [60] when comparing oxidative stability in meat from different forage-based systems and conventional meat. The oxidative stability of meat does not depend solely on tocopherol presence but on a wider complex of interactions between pro- and antioxidant factors [51]. Among antioxidant factors, other dietary compounds, such as phenols and tannins, can play an important role [61–62]. In our study, the higher intake of both tocopherols and total phenols by SPR and SMM groups, relative to WIN, may help explain the enhanced oxidative stability in the meat of the respective season. These findings are further of interest considering the higher myoglobin content in SPR and SMM meats compared to WIN meat. Indeed, the heme iron contained in this protein is a known contributor that may trigger lipid oxidation [63].

Moreover, myoglobin greatly contributes to determining meat color, one of the main factors that drives consumer’s preference for fresh meat. Usually, the greater the myoglobin content, the redder the meat [64]. In this study, a* did not differ between dietary season at d0 despite SPR and SMM meat showed greater myoglobin content than WIN meat. Intrinsic factors such as age, final pH, and fatness that may affect meat color parameters [65] were similar between animals, as they were slaughtered at target weight and showed similar age, carcass dressing and pH. Moreover, meat color was determined after 21-day meat ageing, which could have further affected this result. Indeed, aging may impair beef meat color stability due to several mechanisms related to reduced metmyoglobin reducing activity and mitochondrial functions. Previously, Seyfert et al. [66] observed that an aging time longer than 14 days is detrimental to beef meat color, reporting a 200% decrease in the redness (a*) of beef meat aged for 21 days than 11 days.

Overall, a* as well as h* and C*, deteriorated faster in WIN and SMM groups as compared to SPR. If the differences between WIN and SPR meat could lie in the different intake of fresh herbage during the two seasons [67], both the SPR and SMM animals were mainly fed at grazing. Nevertheless, the proportion among antioxidants differed between SPR and SMM diet. In particular, tannins represented 16% of total phenols in the SPR diet, whereas they accounted for 52% in the SMM diet. Tannins, especially condensed tannins, are less absorbed by the digestive tract of the ruminants than other soluble phenols [68–69], which could have partially reduced the color stability in SMM meat than SPR meat. Indeed, Luciano et al. [70] found that dietary phenols (i.e., isoflavones) can be very effective in protecting meat against oxidative damage. The evolution of L* over storage time in the meat from different seasons deserves further investigation as an uncommon trend was observed for SPR meat. Indeed, a conserved-forage based diet may result in greater meat lightness than a fresh-herbage based diet [43], which aligns with the results of this study. Opposite, in this study, L* in SPR meat showed an increasing trend over time, showing the lowest value at d0 and the greatest after 7 days of storage, when L* is commonly expected to lower or remain stable over refrigerated storage.

Differing from univariate analysis, which focuses on a single dependent variable at a time, assessing its distribution, central tendency, or the effect of one or more predictors, the multivariate approach (e.g., PCA and Linear Discriminant Analysis, as used in this study) simultaneously considers multiple variables to uncover underlying patterns, reduce dimensionality, or classify observations based on their multivariate profiles. In this study, multivariate statistical analysis was employed with two primary objectives: to evaluate whether the multivariate structure of meat quality parameters could reveal overall patterns useful for discriminating the meat according to the seasonal diet, and to identify the parameters with the greatest discriminative power. Only linear discriminant analysis, coupled with a stepwise selection procedure, proved effective in identifying the variables that best differentiated meat based on the feeding seasons. Four of these variables were related to meat fatty acids (17:1 c9, 18:1 *t*10, 22:0; 22:5 n-6), two to oxidative stability (TBARS and Metmyoglobin %), and one to color (b*), in addition to the farming time. The discriminant power of the selected variables was remarkably high, allowing for the correct classification of approximately 90% of the samples into their respective seasonal groups. CAN1, representing the x-axis of the canonical plane, allowed for a distinction of SMM meat from the two other groups, whereas CAN 2 (y-axis of the canonical plane) partially reflected the distribution of samples across the three feeding seasons. Focusing on the variables retained after the stepwise procedure, a direct relationship with feeding season can reasonably be assumed for some of them. This is the case for b*, for which the link between carotenoid intake and meat color is well established [67,71]. Likewise, the selection of TBARS and Metmyoglobin %, both indicators of meat oxidative status [30,71], may reflect seasonal differences in the availability and diversity of dietary antioxidants. In contrast, fatty acid–related variables appear to be less directly associated with feeding season and more strongly influenced by animal metabolism. The presence of 18:1 *t*10 in meat, mainly resulting from ruminal biohydrogenation of polyunsaturated fatty acids [72–73], is known to vary with dietary concentrate levels, a factor that can be excluded in the present study since concentrates were never included in the feeding regime. As for 17:1 *c*9, it originates from the interaction between rumen microbial metabolism, responsible for 17:0 synthesis, and endogenous tissue metabolism mediated by Δ^9^-desaturase activity [72]. Similarly, 22:5 n-6, which arises from the endogenous elongation and desaturation of 18:2 *c*9*c*12 [73], may be indirectly linked to linoleic acid intake. However, the dietary distribution of its precursor does not mirror the separation observed in the canonical plot. Although arising from a limited sample size and having the inherent limitations of a farm-case study, these findings together with the remarkable contribution of several variables to both the CAN1 and CAN2 underline the value of a multivariate approach for interpreting the subtle nature of differences in complex, real-world systems, where variability is distributed across multiple contributing factors and can be captured through integrated metabolic signatures rather than direct dietary markers.

## Conclusion

With the caution due to the survey nature of this study, the results indicate that seasonal variation in the proportion and composition of dietary forages during the last three months before slaughtering minimally affected meat quality traits in grass-fed Angus steers. Differences were reported only for minor meat fatty acids, whereas the n-6/n-3 PUFA ratio always remained within the recommended value and comparable across seasons as well as for other health indexes. Similarly, the *t*10/t11 18:1 ratio was constantly below 1 in all the seasons, confirming the major role of dietary forage in affecting these parameters. Resistance of meat lipid against oxidative deterioration and meat color stability during one week of refrigerated storage partially improved along with the presence of fresh herbage in the finishing diet, probably due to the greater antioxidants intake. In addition, meat color parameters seemed to be affected by the type of antioxidants in fresh forages. Despite the limited overall effect of diet on individual meat quality traits, multivariate statistical analysis of these parameters enabled a clear and highly effective discrimination among the three feeding seasons. However, also in the case of multivariate analysis, it should be emphasized that any generalization should take into account the limited sample size and the inherent limitations of a farm-case study.

## Supporting information

S1 Table Effect of feeding season on meat fat percentage and fatty acids (expressed as % of total fatty acids). (DOCX)

S1 Fig Score plot obtained by plotting the first two components obtained from the principal component analysis of the whole dataset of meat quality parameters and farming time. (DOCX)
