# Correction: Seasonal variation in meat quality of Angus steers raised in a Mediterranean forage-fed system: A farm case study

**DOI:** 10.1371/journal.pone.0348314

**Published:** 2026-04-27

**Authors:** Viviana Bolletta, Valentina Roscini, Emanuele Lilli, Chiara Fodaroni, Jacopo Gabriele Orlando, Valentino Mercati, Bernardo Valenti, Mariano Pauselli

There are errors in the author affiliations. The correct affiliations are as follows:

Viviana Bolletta^1^, Valentina Roscini^1^, Emanuele Lilli^1^, Chiara Fodaroni^1^, Jacopo Gabriele Orlando^2^, Valentino Mercati^2^, Bernardo Valenti^1^, Mariano Pauselli^1^

1 Dipartimento di Scienze Agrarie, Alimentari e Ambientali, University of Perugia, Perugia, Italy, 2 Aboca S.P.A. - Società Agricola, Arezzo, Italy
