# Supplementary material for: Seasonal variation in meat quality of Angus steers raised in a Mediterranean forage-fed system: A farm case study
Source: PLoS One. 2026 Mar 24;21(3):e0344517. doi: 10.1371/journal.pone.0344517 (PMC13012490; doi:10.1371/journal.pone.0344517)
Supplement: S1 Table — (DOCX) [file pone.0344517.s001.docx]

**Seasonal variation in meat quality of Angus steers raised in a Mediterranean forage-fed system: A farm case study.**

**Viviana Bolletta^1^, Valentina Roscini^1^, Emanuele Lilli^1^, Chiara Fodaroni^1^, Jacopo Gabriele Orlando^2^, Valentino Mercati^2^, Bernardo Valenti^2*^, Mariano Pauselli^2^.**

^1^ Dipartimento di Scienze Agrarie, Alimentari e Ambientali, Italy

^2^ Aboca S.P.A. - Società Agricola, Arezzo, Italy

* Corresponding author

e-mail: [bernardo.valenti@unipg.it](mailto:bernardo.valenti@unipg.it) (BV)

**Supporting Information**

**S1 Table. Effect of feeding season on meat fat percentage and fatty acids (expressed as % of total fatty acids).**

|  | **Seasonal Diet*^1^*** | | | **SEM*^2^*** | **P-value*^3^*** |
| --- | --- | --- | --- | --- | --- |
|  | WIN | SPR | SMM |  |  |
| **Fat %** | 2.68 | 2.68 | 2.57 | 0.194 | 0.848 |
| **Fatty Acids (g/100g total fatty acids)** | | |  |  |  |
| **12:0** | 0.06 | 0.09 | 0.10 | 0.01 | 0.360 |
| **12:1 *c*9** | 0.02 | 0.03 | 0.02 | 0.01 | 0.141 |
| **14:0** | 2.27 | 2.63 | 2.45 | 0.12 | 0.466 |
| **14:1 *c*9** | 0.56 | 0.64 | 0.54 | 0.04 | 0.541 |
| **15:0 *iso*** | 0.27 | 0.27 | 0.29 | 0.02 | 0.772 |
| **15:0 *anteiso*** | 0.33 | 0.34 | 0.34 | 0.02 | 0.929 |
| **15:0** | 0.70 | 0.74 | 0.67 | 0.04 | 0.762 |
| **16:0** | 25.54 | 26.21 | 24.59 | 0.35 | 0.158 |
| **16:1 *c*9** | 3.20 | 3.16 | 3.27 | 0.11 | 0.920 |
| **17:0 *iso*** | 0.47 | 0.47 | 0.47 | 0.01 | 0.984 |
| **17:0 anteiso** | 0.79 | 0.79 | 0.78 | 0.03 | 0.990 |
| **17:0** | 1.51 | 1.55 | 1.52 | 0.05 | 0.953 |
| **17:1 c9** | 0.88^b^ | 0.89^b^ | 0.99^a^ | 0.02 | 0.009 |
| **18:0** | 14.96 | 13.06 | 13.81 | 0.42 | 0.179 |
| **18:1 t6-7-8** | 0.04 | 0.06 | 0.05 | 0.01 | 0.225 |
| **18:1 t9** | 0.14 | 0.15 | 0.15 | 0.01 | 0.913 |
| **18:1 t10** | 0.11 | 0.08 | 0.09 | 0.01 | 0.145 |
| **18:1 t11** | 1.57 | 1.40 | 1.61 | 0.09 | 0.514 |
| **18:1 c6** | 0.17 | 0.14 | 0.17 | 0.02 | 0.471 |
| **18:1 c9** | 32.06 | 30.39 | 31.41 | 0.59 | 0.513 |
| **18:1 c11** | 1.95^ab^ | 1.48^b^ | 2.56^a^ | 0.17 | 0.032 |
| **18:1 c12** | 0.25 | 0.17 | 0.49 | 0.01 | 0.152 |
| **18:1 c13** | 0.28 | 0.30 | 0.27 | 0.02 | 0.747 |
| **18:2 t9t12** | 0.05 | 0.06 | 0.05 | 0.01 | 0.729 |
| **18:2 c9t11** | 3.94 | 4.46 | 4.25 | 0.02 | 0.919 |
| **18:2 n-6** | 0.39 | 0.37 | 0.39 | 0.30 | 0.788 |
| **18:3 n-6** | 0.04 | 0.04 | 0.04 | 0.01 | 0.943 |
| **18:3 n-3** | 1.41 | 1.63 | 1.47 | 0.01 | 0.434 |
| **20:0** | 0.14 | 0.11 | 0.24 | 0.01 | 0.410 |
| **20:2 n-6** | 0.05 | 0.07 | 0.06 | 0.01 | 0.131 |
| **20:3 n-6** | 0.34 | 0.40 | 0.36 | 0.04 | 0.727 |
| **20:3 n-3** | 0.04 | 0.31 | 0.14 | 0.07 | 0.274 |
| **20:4 n-6** | 1.14 | 1.10 | 0.83 | 0.13 | 0.580 |
| **20:5 n-3** | 0.29 | 0.39 | 0.40 | 0.05 | 0.579 |
| **22:0** | 0.03 | 0.03 | 0.05 | 0.01 | 0.612 |
| **22:4 n-6** | 0.07 | 0.09 | 0.14 | 0.02 | 0.356 |
| **22:5 n-6** | 0.03 | 0.03 | 0.05 | 0.01 | 0.317 |
| **22:5 n-3** | 0.55 | 0.62 | 0.64 | 0.06 | 0.825 |
| **22:6 n-3** | 0.05 | 0.07 | 0.06 | 0.01 | 0.611 |
| **SFA^4^** | 43.01 | 42.13 | 41.23 | 0.55 | 0.447 |
| **MUFA^5^** | 41.23 | 38.88 | 41.64 | 0.72 | 0.238 |
| **PUFA^6^** | 8.30 | 9.60 | 8.84 | 0.56 | 0.641 |
| **OBCFA^7^** | 4.06 | 4.16 | 4.06 | 0.15 | 0.959 |
| **t10/t11 18:1** | 0.09 | 0.06 | 0.06 | 0.01 | 0.248 |
| **PUFA n-6** | 5.58 | 6.21 | 5.75 | 0.42 | 0.825 |
| **PUFA n-3** | 2.33 | 3.02 | 2.71 | 0.12 | 0.336 |
| **PUFA n-6/n-3** | 2.33 | 2.11 | 2.26 | 0.11 | 0.697 |
| **AI^8^** | 0.71 | 0.77 | 0.71 | 0.03 | 0.507 |
| **TI^9^** | 1.33 | 1.26 | 1.23 | 0.04 | 0.588 |
| **HP-PUFA^10^** | 3.92 | 4.69 | 4.14 | 0.27 | 0.503 |
| **PI^11^** | 21.50 | 23.68 | 21.31 | 1.39 | 0.754 |
| **h/H^12^** | 1.46 | 1.40 | 1.52 | 0.04 | 0.488 |
| 1. Diet: WIN = Hay-based diet, with hay ≥ 80% total ingestion); SPR = Spring Pasture-based diet, with pasture ≥ 80% total ingestion diet; SMM = Summer Pasture-based diet, with pasture ≥ 80% total ingestion diet) 2. SEM: Standard error of mean 3. P-value of the effect of seasonal diet; ^a-b^ Means in the same row with different superscripts significantly differ (P ≤ 0.05) 4. SFA: Sum of Saturated Fatty Acids 5. MUFA: Sum of Monounsaturated Fatty Acids 6. PUFA: Sum of Polyunsaturated Fatty Acids 7. OBCFA: Sum of Odd and Brunched Chain Fatty Acids 8. AI: Atherogenic index, (12:0 + 4 * 14:0 + 16:0) / (MUFA + PUFA n-6 + n-3 PUFA) 9. Thrombogenic index, (14:0 + 16:0 + 18:0) / [(0.5 * 18:1 *c*9) + (0.5 * other MUFA) + (0.5 * n-6 PUFA) + (3 * n-3 PUFA) + (n-6/n-3 PUFA)]   ^10^HP-PUFA: Highly peroxidizable polyunsaturated fatty acids calculated as the sum of PUFA with three or more double bonds;  ^11^PI: Peroxidability index, (∑Dienoic fatty acids) + (∑Trienoic fatty acids*2) + (∑Tetraenoic fatty acids*3) + (∑Pentaenoic fatty acids*4) + (∑Hexaenoic fatty acids*5)  ^12^h/H: hypocholesterolemic to hypercholesterolemic fatty acids ratio (18:1*c*9 + PUFA) / (12:0 + 14:0 + 16:0) | | | | | |
