# Supplementary material for: Seasonal variation in meat quality of Angus steers raised in a Mediterranean forage-fed system: A farm case study
Source: PLoS One. 2026 Mar 24;21(3):e0344517. doi: 10.1371/journal.pone.0344517 (PMC13012490; doi:10.1371/journal.pone.0344517)
Supplement: S1 Fig — (DOCX) [file pone.0344517.s002.docx]

**Seasonal variation in meat quality of Angus steers raised in a Mediterranean forage-fed system: A farm case study.**

**Viviana Bolletta^1^, Valentina Roscini^1^, Emanuele Lilli^1^, Chiara Fodaroni^1^, Jacopo Gabriele Orlando^2^, Valentino Mercati^2^, Bernardo Valenti^2*^, Mariano Pauselli^2^.**

^1^ Dipartimento di Scienze Agrarie, Alimentari e Ambientali, University of Perugia, Italy

^2^ Aboca S.P.A. - Società Agricola, Arezzo, Italy

* Corresponding author

e-mail: [bernardo.valenti@unipg.it](mailto:bernardo.valenti@unipg.it) (BV)

**Supporting information**

**S1 Fig – Score plot obtained by plotting the first two components obtained from the principal component analysis of the whole dataset of meat quality parameters and farming time.**


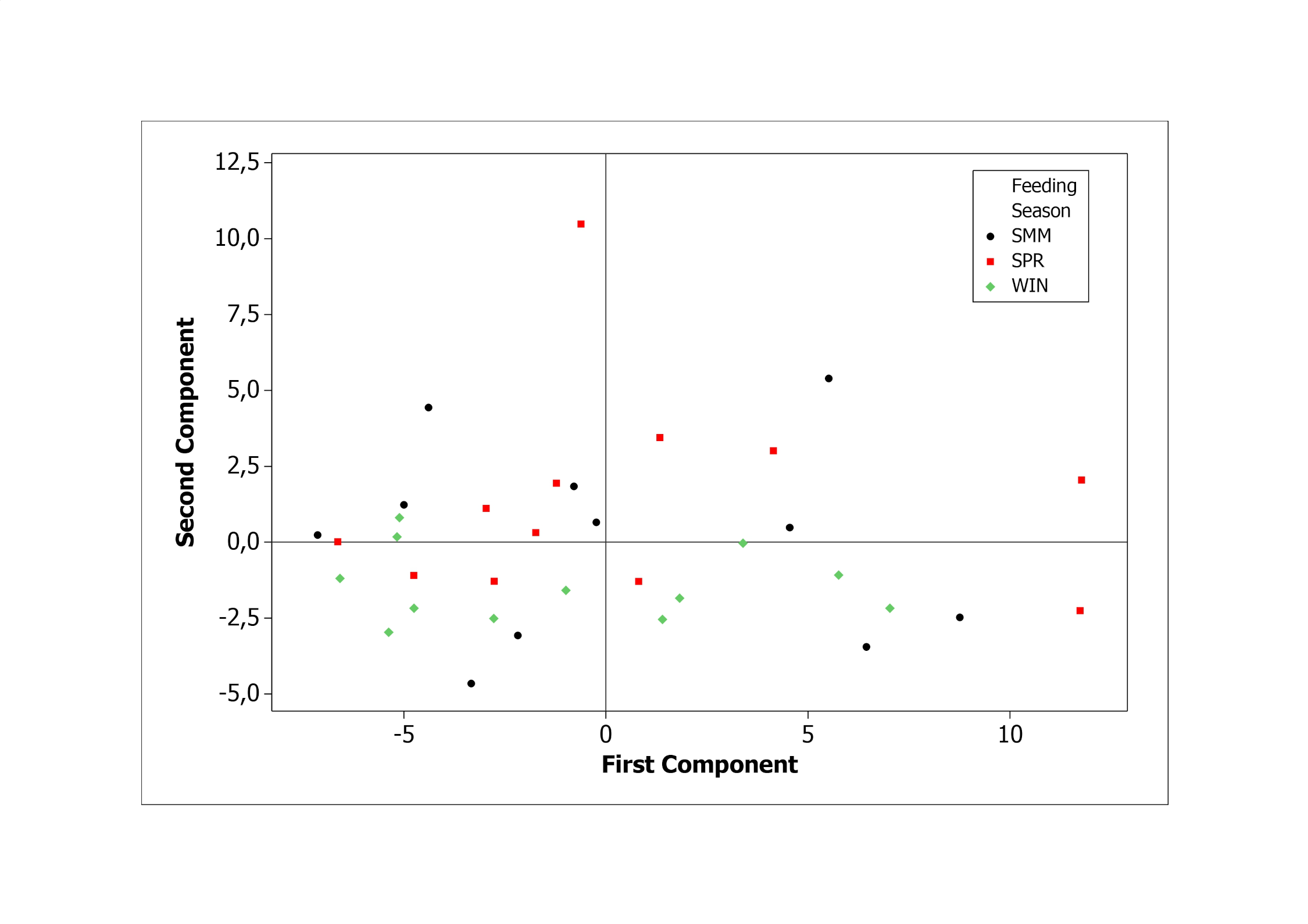


WIN = Hay-based diet, with hay ≥ 80% total ingestion); SPR = Spring Pasture-based diet, with pasture ≥ 80% total ingestion diet; SMM = Summer Pasture-based diet, with pasture ≥ 80% total ingestion diet)
